# Supplementary material for: The genome sequence of a douglasiid moth, Tinagma dryadis Staudinger, 1872 (Lepidoptera: Douglasiidae)
Source: Wellcome Open Res. 2026 Sep 11;11:632. [Version 1] doi: 10.12688/wellcomeopenres.27776.1 (PMC13643139; doi:10.12688/wellcomeopenres.27776.1)
Supplement: Peer review history

# Reviewer report: Reviewer response for version 1

**DOI:** 10.21956/wellcomeopenres.30567.r169150

**Published:** 2026-10-07

**Authors:** Joshua D Gibson

**Affiliations:** 1 Georgia Southern University, Statesboro, Georgia, USA

The genome note “The genome sequence of a douglasiid moth, Tinagma dryadis Staudinger, 1872 (Lepidoptera: Douglasiidae)” by Mutanen et al provides the sequence and assembly of the genome for a previously unsequenced moth species as part Project Psyche. The sequence is based on a single female and utilized a combination of PacBio HiFi long-read sequences and Hi-C sequencing using an Illumina NovaSeq X. The sequences were assembled into the mitochondrial genome and two haplotypes using Hi-C phasing. Haplotype 1 was assembled into 22 chromosome pseudomolecules with a length of 813.92 Mb,. haplotype 2 was a scaffold-level assembly with a length of 692.99 Mb, and the mitochondrial genome assembly had a length of 15.42 Kb. The combined assembly appears to be relatively complete based on *k*-mer and BUSCO tests, though haplotype 2 is less complete than haplotype 1. All data is freely available and Table 1 provides a very easy overview of the appropriate accessions. Similarly, all software sources are clearly provided and the standardized protocols are adequately provided. The note provides some details of this species and the rationale for sequencing: it is an enigmatic species with poorly resolved phylogenetic relationships and it has an interesting two-year life cycle that is representative of many Arctic-alpine Lepidoptera, which may indicate a potential for this sequence data to help identify a molecular basis for this natural history trait.

 My only minor notes for potential improvement are some clarity on the differences in the two assembled haplotypes. I assume that these represent the two diploid chromosome sets, but this is never explicitly stated as the hypothesized reason for there being two haplotypes. Haplotype 1 contains the W and Z sex chromosomes, does this explain the assembly differences, in particular the BUSCO score differences? If so, how much of this is expected based on the BUSCO genes found on these chromosomes? This wouldn’t require extensive editing, but addressing these more explicitly would make the genome note more accessible by connecting some of the genome details to the biology of the moth.

Are the datasets clearly presented in a usable and accessible format, and the assembly and annotation available in an appropriate subject-specific repository?

Yes

Are sufficient details of the sequencing and extraction, software used, and materials provided to allow replication by others?

Yes

Are the rationale for sequencing the genome and the species significance clearly described?

Yes

Are the protocols appropriate and is the work technically sound?

Yes

Reviewer Expertise:

Evolutionary genetics and genomics, insect physiology, social insect biology

I confirm that I have read this submission and believe that I have an appropriate level of expertise to confirm that it is of an acceptable scientific standard.

# Reviewer report: Reviewer response for version 1

**Published:** 2026-09-11

**Authors:** Automated Benchmarking

**Affiliations:** 1 Wellcome Sanger Institute, Hinxton, UK

As this article is a Genome Note, Automated Benchmarking has been used to determine whether the reported assembly passes three defined checks established by the Earth BioGenome Project: the raw and assembled data are publicly available, the Earth BioGenome Project three-part metric is met, and the BUSCO score passes the stated threshold. For Tinagma dryadis (ilTinDrya1), all three checks passed. Data available: pass. ENA study PRJEB97050, raw-read accessions ERR15532278 and ERR15551580, and assembly accessions GCA_977019765.1 and GCA_977019915.1 are public. Earth BioGenome Project three-part metric: pass. Haplotype 1 is reported as 7.C.Q69, meeting the 6.C.Q40 reference standard. BUSCO: pass. Haplotype 1, which is proposed as the reference assembly for this species, has 98.1% complete BUSCOs (97.0% single-copy and 1.1% duplicated). It meets the target of greater than 90% complete and single-copy BUSCOs. This decision uses values reported in the Genome Note and public accession records.

Reviewer Expertise:

NA

I confirm that I have read this submission and believe that I have an appropriate level of expertise to confirm that it is of an acceptable scientific standard.
